# Editorial for the research topic: information-based methods for neuroimaging: analyzing structure, function and dynamics

**DOI:** 10.3389/fninf.2014.00086

**Published:** 2014-12-19

**Authors:** Jesus M. Cortes, Daniele Marinazzo, Miguel A. Muñoz

**Affiliations:** ^1^Biocruces Health Research Institute, Hospital Universitario CrucesBarakaldo, Spain; ^2^Ikerbasque: The Basque Foundation for ScienceBilbao, Spain; ^3^Data Analysis, University of GentGent, Belgium; ^4^Departamento de Electromagnetismo y Física de la Materia and Instituto Carlos I de Física Teórica y Computacional, University of GranadaGranada, Spain

**Keywords:** information theory, brain connectivity, network dynamics, neuroimaging, neuroinformatics, Granger causality, entropy, mutual information

This Research Topic gathers different contributions highlighting novel types of analysis and methods to deal more efficiently with neuroimaging data, simulated and real, acquired with different modalities. These approaches allow us to shed light on the mechanisms of brain organization, with focus on the relationship between brain structure, function and dynamics.

The first article of this Topic (Sanz Leon et al., 2013), introduces The Virtual Brain, a Neuroinformatics platform for full brain network simulations using realistic connectivity, putting in evidence that the integration between brain structure and function is perfectly plausible by simulating realistic brain activity (more specifically, neural mass models) on the architecture of the structural connectome. The authors show that dynamical models aimed at reproducing the functional connectivity patterns observed in the resting brain exhibit a much better performance when they are tuned around a balanced state favoring the shifting between attractors. This balanced state is described in terms of energy landscape, as discussed also in Watanabe et al. (2014), where the state transitions (in terms of energy landscape) between two representative Resting State Networks—the Default Mode Network and the Fronto-Parietal Network—are addressed.

With an alternative approach, the relation between structural and functional networks is tackled in Ajilore et al. (2013), by using the functional-by-structural hierarchical (FSH) mapping. This was developed for multimodal integration of the resting state fMRI (rsfMRI) and the whole brain (tractography-derived) connectome and is based on the evidence that the level of resting-state functional correlation between any two regions (in general) decreases as the graph distance of the corresponding structural connectivity matrix between them increases. Results are reported in health and depression.

Effective connectivity methods are devised to infer directed connectivity patterns from time-series data. A new method based on Variational Bayesian Inference to infer causality from time series was proposed in Luessi et al. (2014). The method uses a vector autoregressive model for the latent variables describing neuronal activity in combination with a linear observation model based on a convolution with a hemodynamic response function. The method is validated using both real and synthetic resting fMRI data.

Continuing with the problem of causality inference, classical methods like Granger causality were extended to the situation of time-varying signals in Chicharro and Panzeri (2014). This study also provides a graphical approach to predict dynamic statistical dependencies between the signals from the causal structure.

A different approach is presented in Kolchinsky et al. (2014), where brain regions and networks are characterized by information-theoretic measures using both functional and structural information in a complementary manner. In particular, Kolchinsky et al., quantify the amount of functional coupling between sets of regions of interest (ROIs) as well as integration within sets of ROIs. Several information-based measures are considered, and their scaling with subsystem size is explored.

Regarding consciousness and its relationship with Information Theory, two papers have been contributed to this Research Topic. In Lee et al. (2013) the Approximate Entropy (ApEn), a measure known to correlate with the level of brain consciousness, is used to characterize EEG signals in children and adults to show that the amount of ApEn is lower in children and that it correlates, in children as in adults, with consciousness; in particular, the authors show that ApEn decreases across the transition from awake to REM sleep to non-REM sleep. For patients with deficit of consciousness (DOC) after traumatic brain injury, (Mäki-Marttunen et al., 2013) show two possible markers from fMRI time series that can distinguish between DOC patients and healthy subjects. The inter-hemispheric correlations (but not the intra-hemispheric correlations) between left-right homolog areas decrease, as does the intra-hemispheric information flow, in DOC patients compared to control. For a small group of 4 patients who fully recovered from coma, the study also reports an increase of the intra-hemisphere information flow with respect to controls.

Information-based measures have been associated with altered information processing in Autism Spectrum Disorder (ASD). The close link between active information storage and general theories of cortical function has been addressed in Gómez et al. (2014), by analyzing magnetoencephalography (MEG) signals. The authors report a significant reduction of information storage in the hippocampus in ASD patients. The amount of information and entropy of MEG signals in ASD (Asperger syndrome in this case) has been analyzed also in Pérez Velázquez and Galán (2013). The analysis, carried out at the source level, addresses the relationship between resting state activity and the brain inner processing with regards to information production, as quantified by the relative entropy. The results suggest that the brains of individuals with ASD produce more information than the age-matched participants.

The complementary role of the three components of information processing, transfer, storage and modification is investigated in Wibral et al. (2014). Local information storage is analyzed in detail in neural data and associated to neural properties such as stimulus preferences and surprise.

The connection between information content of brain activity signals and function has been explored also in Sokunbi (2014). The author investigates the power of a similarity measure (the Sample Entropy) to discriminate between young and elderly subjects emphasizing on the possible limitations arising from the reduced length of time series that are commonly encountered in fMRI studies.

Information-based measures are also useful for developing new technical tools for structural and functional analysis. Novel algorithms have been proposed to improve the construction of the structural connectome in Roine et al. (2014), by investigating the isotropic partial volume effects caused by non-white matter tissue on fiber orientation diffusion estimated with constrained spherical de-convolution. Diffusion weighted signals are simulated with varying diffusion weightings, signal-to-noise ratios, fiber configurations, and tissue fractions.

The equivalence between the information-based and model-based approaches to directed dynamical connectivity in the frequency domain was explored in Takahashi et al. (2014). To enhance the understanding of the possibly complex interaction between multiple time series the authors decompose the established approaches to Directed Coherence into different modes of interaction.

Concerning improvements to functional analysis, a novel information-theoretic approach for spatial components ranking has been proposed in Ossadtchi et al. (2013). The proposed method is based on the Mutual Information (MI) Spectrum which serves as a power-invariant measure of repetitive task-related signal in the temporal loadings of spatial components. Using realistic simulations, the authors in show that the task-relatedness measure, based on estimating the MI between a component and the expanded binary stimulus signal, allows for significantly higher detector characteristics when compared with conventional alternatives. The application of the MI Spectrum for the selection of task-related independent components is validated with real MEG data.

We hope that the reader will find in this Research Topic a useful reference for the state of the art in the emerging field of tools rooted in information theory and applied to neuroscience.

## Conflict of interest statement

The authors declare that the research was conducted in the absence of any commercial or financial relationships that could be construed as a potential conflict of interest.
